# Policy Series: Family Caregiving Policies: Where We Are Now

**DOI:** 10.1093/geroni/igab046.242

**Published:** 2021-12-17

**Authors:** Pamela Nadash, Rani Snyder, Eileen Tell

## Abstract

This session reviews prospects for advancing family caregiving policy under the Biden Administration, by reporting on the RAISE (Recognize, Assist, Include, Support, and Engage) Family Caregivers Act, enacted in January 2018. The Act directs the Secretary of HHS to develop a national family caregiving strategy, and supports research and consensus-building activities, in collaboration with The John A. Hartford Foundation. It aims to identify actions that communities, providers, government, and others may take to recognize and support family caregivers. To this end, the Administration for Community Living (ACL) has convened an Advisory Council, comprising 15 voting members from various stakeholder groups, to guide the effort; the project also commissioned primary data collection on caregiver priorities and recommendations, using a Request for Information (RFI) in the Federal Register garnering roughly 1600 responses, 12 focus groups with diverse family caregivers, and listening sessions with stakeholder groups. Wendy Fox-Grage, of the National Academy on State Health Policy, which supports RAISE Act activities, will describe the project’s scope of work and activities to date. Pamela Nadash from the LeadingAge LTSS Center @UMass Boston, who leads the data analysis component, will present findings from the commissioned research, while Molly Evans, (MA Executive Office of Elder Affairs) will review the current state of state-level policies aimed at supporting family caregivers. The symposium will conclude with Grace Whiting, CEO of the National Alliance for Caregiving, who will present an advocate’s perspective on the status of family caregiving policy. Eileen Tell, of ET Consultants, will act as discussant.

